# Evaluation of eravacycline susceptibility testing by MIC test strip and disk diffusion against broth microdilution in carbapenem-resistant Enterobacterales

**DOI:** 10.3389/fcimb.2026.1932734

**Published:** 2026-09-18

**Authors:** Kangyu Wang, Jinjing Tian, Tingchao Wu, Chunzhong Dong, Xinying Wang, Qiang Feng, Zhijun Zhang

**Affiliations:** 1 Tumor Research and Treatment Center, Shandong Provincial Hospital Affiliated to Shandong First Medical University, Jinan, China; 2 Postdoctoral Research Station, The Affiliated Taian City Central Hospital of Qingdao University, Taian, China; 3 Department of Laboratory Medicine, Liaocheng Second People’s Hospital, Liaocheng, China; 4 Department of Laboratory Medicine, The Affiliated Taian City Central Hospital of Qingdao University, Taian, China; 5 Department of Laboratory Medicine, Binzhou People’s Hospital, Binzhou, China; 6 Shandong Provincial Key Medical and Health Laboratory of Anti-Drug Resistant Drug Research, The Affiliated Taian City Central Hospital of Qingdao University, Taian, China

**Keywords:** broth microdilution, carbapenem-resistant Enterobacterales, disk diffusion, eravacycline, MIC test strip

## Abstract

**Objectives:**

Eravacycline is a novel fluorocycline with potent activity against carbapenem-resistant Enterobacterales (CRE). However, comparative validation of gradient diffusion (MIC Test Strip, MTS) and disk diffusion (DD) against reference broth microdilution (BMD) specifically in CRE isolates remains limited, particularly under region-specific breakpoint systems.

**Methods:**

This study evaluated the performance of the MTS and DD against BMD for eravacycline susceptibility testing in 358 CRE clinical isolates (174 carbapenem-resistant *Klebsiella pneumoniae*, CRKP and 144 carbapenem-resistant *Escherichia coli*, CREC), using both U.S. Food and Drug Administration (FDA) and Expert Committee of the National Health Commission on Antimicrobial Susceptibility Testing and Standard Research (ChinaCAST) breakpoints. Categorical agreement (CA), essential agreement (EA), major error (ME), and very major error (VME) were assessed according to CLSI M52 criteria, and Bland-Altman analysis was performed for quantitative agreement between MTS and BMD.

**Results:**

BMD showed MIC_50_/MIC_90_ of 0.25/1 mg/L, with overall susceptibility rate of 81.2% by FDA breakpoints. For MTS, overall CA and EA were 92.8% and 92.5%, but VME was 33.8% under FDA breakpoints, concentrated in CRKP (35.5%). Under ChinaCAST breakpoints, CRKP CA increased to 94.8%, yet VME decreased to 18.2% and ME increased to 4.3%. For DD, overall CA was 89.9% with VME of 32.3%; only CREC under FDA breakpoints achieved acceptable CA (97.9%) and error rates, whereas ChinaCAST breakpoints yielded unacceptable CA (77.0-77.8%) and elevated ME (22.4-24.5%) for both species.

**Conclusions:**

Eravacycline retains potent *in vitro* activity against CRE. MTS is suitable for routine testing of CREC, and DD is reliable for CREC under FDA breakpoints. For CRKP, neither method satisfied CLSI M52 criteria, and reference BMD remains indispensable.

## 1 Introduction

Antimicrobial resistance has emerged as a formidable global public health challenge, with the 2021 Global Burden of Disease study estimating approximately 4.71 million deaths attributable to bacterial antimicrobial resistance (Macesic et al., 2025). Carbapenem-resistant *Enterobacterales* (CRE), as key representatives of multidrug-resistant Gram-negative pathogens, constitute a major driver of this burden, with associated mortality elevated, hospital stays prolonged, and healthcare costs increased (Cassini et al., 2019; Temkin et al., 2024). The World Health Organization has designated CRE as highest-priority resistant pathogens (Perez and Bonomo, 2019; Sati et al., 2025). As one of the largest consumers of antimicrobial agents globally, China faces a particularly alarming CRE epidemic; according to 2025 CHINET surveillance data, the meropenem resistance rate in *Klebsiella pneumoniae* has reached 21.0%, representing a marked increase from 15.0% in 2015 (China Antimicrobial Surveillance Network, 2026). However, effective therapeutic options for CRE infections remain limited. Traditional last-resort agents, polymyxins and tigecycline, are restricted by nephrotoxicity and poor tissue penetration (Macesic et al., 2025). Although novel β-lactam/β-lactamase inhibitor combinations, including ceftazidime-avibactam, aztreonam-avibactam, and imipenem-relebactam, have expanded treatment options (Ma et al., 2023), eravacycline, a novel fully synthetic fluorocycline approved by the U.S. Food and Drug Administration (FDA) in 2018 and Chinese authorities in 2023, offers a distinct therapeutic alternative. Through fluorine substitutions at the C-7 and C-9 positions, eravacycline overcomes efflux pump and ribosomal protection-mediated resistance, with *in vitro* activity superior to tigecycline against CRE producing KPC, NDM, and OXA-48 (Sutcliffe et al., 2013; Solomkin et al., 2017; Heaney et al., 2019). *In vitro* studies report eravacycline MIC_50_/_90_ values for carbapenem-resistant *E. coli* (CREC) of 0.25/0.5 mg/L; the corresponding values for carbapenem-resistant *K. pneumoniae* (CRKP) are 0.5–1/1–2 mg/L (Zhang et al., 2016; Zhao et al., 2019; Alosaimy et al., 2020). Eravacycline has been recommended for CRE infections by the Infectious Diseases Society of America (IDSA) and the European Society of Clinical Microbiology and Infectious Diseases (ESCMID) (Paul et al., 2022; Tamma et al., 2024). Accurate susceptibility testing is therefore essential for guiding eravacycline therapy in CRE infections.

Broth microdilution (BMD) serves as the reference method for *in vitro* eravacycline susceptibility testing; however, its stringent technical requirements, protracted procedures, and high cost limit its widespread adoption in routine clinical laboratories. Disk diffusion (DD) and gradient diffusion methods offer operational simplicity and reduced cost, making them suitable for individual isolate testing and widely applicable in most microbiology laboratories. Furthermore, international harmonization of eravacycline susceptibility breakpoints remains incomplete. The FDA has established breakpoints for Enterobacterales covering *Escherichia coli (E*. *coli)*, *K. pneumoniae*, *Citrobacter freundii*, *Klebsiella oxytoca*, and *Enterobacter cloacae* (U.S. Food and Drug Administration, 2022 [Internet]). European Committee on Antimicrobial Susceptibility Testing (EUCAST) has established a susceptible breakpoint of ≤0.5 µg/mL for *E. coli* only, which aligns with both FDA and ChinaCAST breakpoints (The European Committee on Antimicrobial Susceptibility Testing (EUCAST), 2026). However, the Clinical and Laboratory Standards Institute (CLSI) has not yet issued official breakpoints for this agent (Clinical and Laboratory Standards Institute (CLSI), 2026). In China, the Expert Committee of the National Health Commission on Antimicrobial Susceptibility Testing and Standard Research (ChinaCAST) published breakpoints in 2025 exclusively for *E*. *coli* and *K. pneumoniae* (National Health Commission Expert Committee on Clinical Antimicrobial Susceptibility Breakpoint et al., 2025), which are precisely the predominant pathogens in Chinese CRE infections and account for the vast majority of CRE isolates. Although this targeted strategy aligns with Chinese epidemiological features, comparative validation of gradient diffusion and disk diffusion against reference BMD specifically in CRE isolates remains limited, particularly when region-specific breakpoints (FDA and ChinaCAST) are applied. We compared the performance characteristics of gradient diffusion (MIC Test Strip, MTS) and DD against BMD for eravacycline susceptibility testing in CRE clinical isolates, aiming to provide an evidence-based reference for method selection, breakpoint interpretation, and clinical decision-making under both FDA and ChinaCAST breakpoints.

## 2 Materials and methods

### 2.1 Bacterial isolates

A total of 358 CRE clinical isolates were collected from the Affiliated Tai’an Central Hospital of Qingdao University. Species identification was confirmed by MALDI-TOF MS (Autof ms1000; Autobio, China). During strain collection, only the first isolate from the same site of each patient was retained. The collection comprised predominantly *K. pneumoniae* (n=174, 48.6%) and *E. coli* (n=144, 40.2%), along with 40 isolates (11.2%) of other Enterobacterales including *Enterobacter cloacae*, *Raoultella ornithinolytica*, *Klebsiella oxytoca*, and other species. CRE status was defined according to CLSI M100 criteria (Clinical and Laboratory Standards Institute (CLSI), 2026): ertapenem MIC ≥2 mg/L or imipenem/meropenem MIC ≥4 mg/L; for Proteus and Providencia species with intrinsic reduced imipenem susceptibility, other carbapenems were used. Carbapenemase genotypes were determined by whole-genome sequencing (WGS). Among the 174 CRKP isolates, *bla*_KPC-2_ predominated (137, 78.7%), with *bla*_NDM-1_ in 7 (4.0%), *bla*_NDM-5_ in 2 (1.1%), *bla*_KPC-3_ in 1 (0.6%), *bla*_NDM-23_ in 1 (0.6%), *bla*_KPC-2_ and *bla*_NDM-1_ co-harbored in 6 (3.4%), and no known carbapenemase genes in 20 (11.5%). Among the 144 CREC isolates, *bla*_NDM-5_ was dominant (117, 81.3%), followed by *bla*_NDM-1_ in 9 (6.3%), *bla*_NDM-13_ in 3 (2.1%), *bla*_NDM-9_ in 3 (2.1%), *bla*_KPC-2_ in 1 (0.7%), *bla*_KPC-2/NDM-1_ co-harbored in 3 (2.1%), and no known carbapenemase genes in 7 (4.9%). The remaining 40 isolates comprised *Enterobacter cloacae* (n=22), *Raoultella ornithinolytica* (n=5), and other species (n=13). All carbapenemase profiles are detailed in **Table 1**. Isolates were recovered from blood (52.0%, n=186), sterile body fluids excluding blood (19.6%, n=70), urine (13.7%, n=49), sputum (8.9%, n=32), and other sources (5.9%, n=21). All isolates were verified, suspended in brain-heart infusion broth with 15% glycerol, and stored at -80 °C until tested. Prior to each susceptibility assay, isolates were sub-cultured twice on sheep-blood agar to ensure purity and viability.

**Table 1:** Carbapenemase genotypes of the 358 CRE clinical isolates.

Species	*bla*KPC-2	*bla*NDM-5	*bla*NDM-1	*bla*NDM-4	blaNDM-13	*bla*NDM-9	blaKPC-3	blaNDM-23	*bla*NDM-6	NO	blaKPC-2+ blaNDM-1	Total
*K. pneumoniae*	137	2	7	0	0	0	1	1	0	20	6	174
*E. coli*	1	117	9	0	3	3	0	0	1	7	3	144
*E. cloacae*	0	7	15	0	0	0	0	0	0	0	0	22
*R. ornithinolytica*	1	0	0	4	0	0	0	0	0	0	0	5
*K. oxytoca*	0	0	0	3	0	0	0	0	0	0	0	3
*K. aerogenes*	1	0	0	0	0	0	0	0	0	1	0	2
*C. freundii*	0	0	0	1	0	0	0	0	0	1	0	2
*P. rettgeri*	0	0	0	2	0	0	0	0	0	0	0	2
*P. mirabilis*	0	0	1	0	0	0	0	0	0	0	0	1
*Salmonella* spp.	0	0	0	0	0	0	0	0	0	1	0	1
*S. marcescens*	0	0	0	2	0	0	0	0	0	0	0	2
Total	140	126	32	12	3	3	1	1	1	30	9	358

NO, no known carbapenemase gene detected.

### 2.2 Whole-genome sequencing and resistance gene identification

Fresh CRE colonies were used for DNA extraction (Bacterial DNA Kit, OMEGA) and whole-genome sequencing (Illumina NovaSeq platform, Shanghai Biozeron Biological Technology Co., Ltd.). Genome assembly was conducted using ABySS, and acquired antimicrobial resistance genes, including carbapenemase genes, were identified from assembled contigs using ResFinder v4.7 (Center for Genomic Epidemiology).

### 2.3 Antimicrobial susceptibility testing reagents and methods

Eravacycline susceptibility was determined in parallel by BMD, MTS, and DD. The MTS (0.002–32 mg/L) and disks (20 μg) were obtained from Liofilchem (Italy). The MTS was FDA-cleared for eravacycline susceptibility testing; the disks were not FDA-cleared at the time of this evaluation. For BMD, eravacycline powder was provided by Everest Medicines (China) and serially twofold diluted in cation-adjusted Mueller-Hinton broth (CAMHB; Becton Dickinson, USA) to achieve final concentrations of 0.015–32 mg/L. A 0.5 McFarland suspension (approximately 1×10^8^ CFU/mL) was prepared. For BMD, the suspension was diluted 1:100 in CAMHB to approximately 1×10^6^ CFU/mL. Fifty microliters of this diluted inoculum were added to each 50 µL drug-containing well, yielding a final concentration of approximately 5×10^5^ CFU/mL. For MTS and DD, the undiluted suspension was applied to Mueller–Hinton agar plates (MH; Zhengzhou, China) within 15 minutes. MTS strips and disks were placed on the same plate with adequate separation to prevent overlapping inhibition zones, and the same inoculum was used to inoculate drug-containing CAMHB microdilution plates. All MH agar plates and microdilution plates were incubated at 35 ± 2 °C in ambient air for 16-20 hours. BMD served as the reference method and was performed according to CLSI M07 (Clinical and Laboratory Standards Institute (CLSI), 2024a); the MIC endpoint was defined as the lowest drug concentration showing no visible growth, explicitly ignoring pinpoint microcolonies and hazy trailing growth per the Chinese national guideline (National Health Commission Expert Committee on Clinical Antimicrobial Susceptibility Breakpoint et al., 2025). DD and MTS were performed in accordance with CLSI M02 (Clinical and Laboratory Standards Institute (CLSI), 2024b) and the manufacturer’s instructions. MTS MIC values corresponding to 80% growth inhibition were recorded; values falling between standard twofold dilutions were rounded up to the next higher twofold concentration before interpretation. Susceptibility categorization followed FDA and ChinaCAST breakpoints (U.S. Food and Drug Administration, 2022 [Internet]; National Health Commission Expert Committee on Clinical Antimicrobial Susceptibility Breakpoint et al., 2025) (**Table 2**). Isolates yielding discordant categorical results (ME or VME) between BMD and MTS/DD were retested by BMD in duplicate to confirm the reference MIC. Quality control was performed in each testing session using *E. coli* ATCC 25922, with all results falling within the acceptable ranges specified by CLSI M100 (Clinical and Laboratory Standards Institute (CLSI), 2026).

**Table 2:** Eravacycline susceptibility breakpoints for Enterobacteriaceae defined by FDA and ChinaCAST.

Organism	ChinaCAST breakpoints				FDA breakpoints^a^			
	MIC(mg/L)		DD (mm)		MIC(mg/L)		DD (mm)	
	S	R	S	R	S	R	S	R
*E. coli*	≤0.5	–	≥17	–	≤0.5	–	≥15	–
*K. pneumoniae*	≤1	–	≥15	–	≤0.5	–	≥15	–
Enterobacteriaceae	–	–	–	–	≤0.5	–	≥15	–

S, Susceptible; R, Resistant; MIC, Minimum Inhibitory Concentration; ChinaCAST, Expert Committee of the National Health Commission on Antimicrobial Susceptibility Testing and Standard Research; FDA, Food and Drug Administration; “-” indicates no breakpoint has been defined for the corresponding organism and test method by the respective standardizing body; “a”Clinical efficacy was shown for *Citrobacter freundii, Enterobacter cloacae, Escherichia coli, Klebsiella oxytoca, Klebsiella pneumoniae*.

### 2.4 Data analysis

Methodological validation was performed according to CLSI M52 (Clinical and Laboratory Standards Institute (CLSI), 2015), using BMD as the reference method to evaluate MTS and disk diffusion performance in terms of: (1) categorical agreement (CA), defined as concordant susceptible/nonsusceptible categorization; (2) essential agreement (EA), defined as MIC values within ±1 twofold dilution of the reference; (3) major error (ME, false resistance); and (4) very major error (VME, false susceptibility). For agents without an intermediate interpretive category, VMEs and MEs occurring within EA were excluded from adjusted calculations per FDA STMA guidance (Food and Drug Administration, 2015), as previously applied in eravacycline method-validation studies (Blanchard et al., 2023). Acceptability criteria were EA and CA ≥90%, ME ≤3.0%, and VME ≤1.5%. Of the 358 CRE isolates, 345 with FDA- or ChinaCAST-defined breakpoints were included in categorical agreement and Bland-Altman analyses; species-specific CA/EA/VME/ME calculations were restricted to CRKP (n=174) and CREC (n=144) due to limited sample sizes for other breakpoint-applicable species (22 *Enterobacter cloacae*, 3 *Klebsiella oxytoca*, and 2 *Citrobacter freundii*). The remaining 13 isolates lacking applicable breakpoints were included only in MIC distribution analysis (**Table 3**). Bland-Altman analysis was used to assess quantitative MIC agreement between MTS and BMD. Because antimicrobial MICs follow a twofold dilution scale, raw values were log_2_-transformed to calculate the difference (d = log_2_MTS − log_2_BMD) and mean between methods. The bias and its 95% confidence interval (CI) were then determined. All analyses were performed using GraphPad Prism 10.4.2.

**Table 3:** Eravacycline MICs and inhibition zone diameters of 13 CRE isolates excluded from categorical agreement analysis due to absence of FDA and ChinaCAST breakpoints.

NO.	BMD (mg/L)	MTS (mg/L)	DD (mm)	Carbapenemase	Species
156	0.5	0.25	16	*bla*_NDM-4_	*S. marcescens*
161	0.5	0.5	16	*bla*_NDM-4_	*S. marcescens*
114	2	4	13	NO	*Salmonella*
235	1	1	15	*bla*_NDM-4_	*P. mirabilis*
112	0.25	2	18	*bla*_NDM-4_	*P. rettgeri*
190	1	1	16	*bla*_NDM-4_	*P. rettgeri*
96	0.25	0.064	20	*bla*_KPC-2_	*R. ornithinolytica*
127	0.125	0.064	20	*bla*_NDM-4_	*R. ornithinolytica*
130	0.125	0.125	19	*bla*_NDM-4_	*R. ornithinolytica*
132	0.125	0.064	19	*bla*_NDM-4_	*R. ornithinolytica*
138	0.25	0.064	17	*bla*_NDM-4_	*R. ornithinolytica*
54	0.25	0.25	17	NO	*K. aerogenes*
208	0.5	0.25	17	*bla*_KPC-2_	*K. aerogenes*

BMD, broth microdilution; MTS, MIC Test Strip; DD, disk diffusion; NO, no known carbapenemase gene detected.

## 3 Results

### 3.1 Broth microdilution

BMD revealed that eravacycline MICs for the 358 CRE isolates ranged from 0.06 to 4 mg/L, with MIC_50_ and MIC_90_ values of 0.25 and 1 mg/L, respectively. Under FDA breakpoints, the overall susceptibility rate among 345 isolates was 81.2% (280/345). By species, 174 CRKP isolates showed a susceptibility rate of 64.4% (112/174), with MIC_50_ and MIC_90_ of 0.5 and 1 mg/L, respectively; 144 CREC isolates showed a susceptibility rate of 99.3% (143/144), with MIC_50_ and MIC_90_ of 0.25 and 0.5 mg/L, respectively (**Table 4**). The remaining 13 isolates lacked applicable breakpoints, and their MIC distributions are presented in **Table 3**.

**Table 4:** MIC distribution of eravacycline against CRE (BMD, mg/L).

Organism	No.of isolates with the eravacycline MIC (mg/L)						
	0.06	0.125	0.25	0.5	1	2	4
*K. pneumoniae*	0	8	36	68	51	11	0
*E. coli*	7	56	60	20	0	1	0
*E. cloacae*	0	0	11	9	1	0	1
*R. ornithinolytica*	0	3	2	0	0	0	0
*K. oxytoca*	0	1	1	1	0	0	0
*C. freundii*	0	0	2	0	0	0	0
*P. rettgeri*	0	0	1	0	1	0	0
*S. marcescens*	0	0	0	2	0	0	0
*K. aerogenes*	0	0	1	1	0	0	0
*P. mirabilis*	0	0	0	0	1	0	0
*Salmonella*	0	0	0	0	0	1	0

BMD, Broth microdilution; MIC, Minimal Inhibitory Concentration.

Among the 65 isolates with BMD MIC >0.5 mg/L under FDA breakpoints, 62 were *K. pneumoniae*, 2 were *E. cloacae*, and 1 was *E. coli*. Carbapenemase genotyping revealed *bla*_KPC-2_ in 45 (69.2%), carbapenemase-negative in 10 (15.4%), *bla*_NDM_ variants in 6 (9.2%), and other genotypes in 4 (6.2%) (**Supplementary Table S1**). Screening for known eravacycline resistance determinants identified *tet*(X4) in one carbapenemase-negative *E. coli* isolate (BMD MIC = 2 mg/L); classic tetracycline resistance genes *tet*(A) and *tet*(D) were present in 18 and 2 isolates, respectively.

### 3.2 MIC test strip

The MTS testing yielded eravacycline MICs ranging from 0.032 to 8 mg/L, with MIC_50_ and MIC_90_ values of 0.25 and 1 mg/L, respectively. Using FDA breakpoints, the susceptibility rate among 345 CRE isolates was 86.7% (299/345), with CA and EA of 92.8% (320/345) and 92.5% (319/345), respectively. Bland-Altman analysis of log_2_-transformed MICs showed a bias of -0.29, a standard deviation of 0.86, and 95% limits of agreement from −1.98 to +1.40. The negative bias indicates that MTS tended to yield slightly lower MIC values than BMD (**Figure 1**). Compared with BMD under FDA breakpoints, ME and VME for all 345 CRE isolates were 1.1% (3/280) and 33.8% (22/65), respectively. After excluding VMEs occurring within EA per FDA STMA guidance, the adjusted VME rate was 9.2% (6/65). Stratified by species, CRKP showed CA of 87.4% (152/174) and VME of 35.5% (22/62), with an adjusted VME of 9.7% (6/62), whereas CREC showed CA of 100.0% (144/144) with no VME. Under ChinaCAST breakpoints, CRKP CA increased to 94.8% (165/174), and the unadjusted VME decreased to 18.2% (2/11), with an adjusted rate of 9.1% (1/11) (**Figure 2**, **Table 5**).

**Figure 1:**
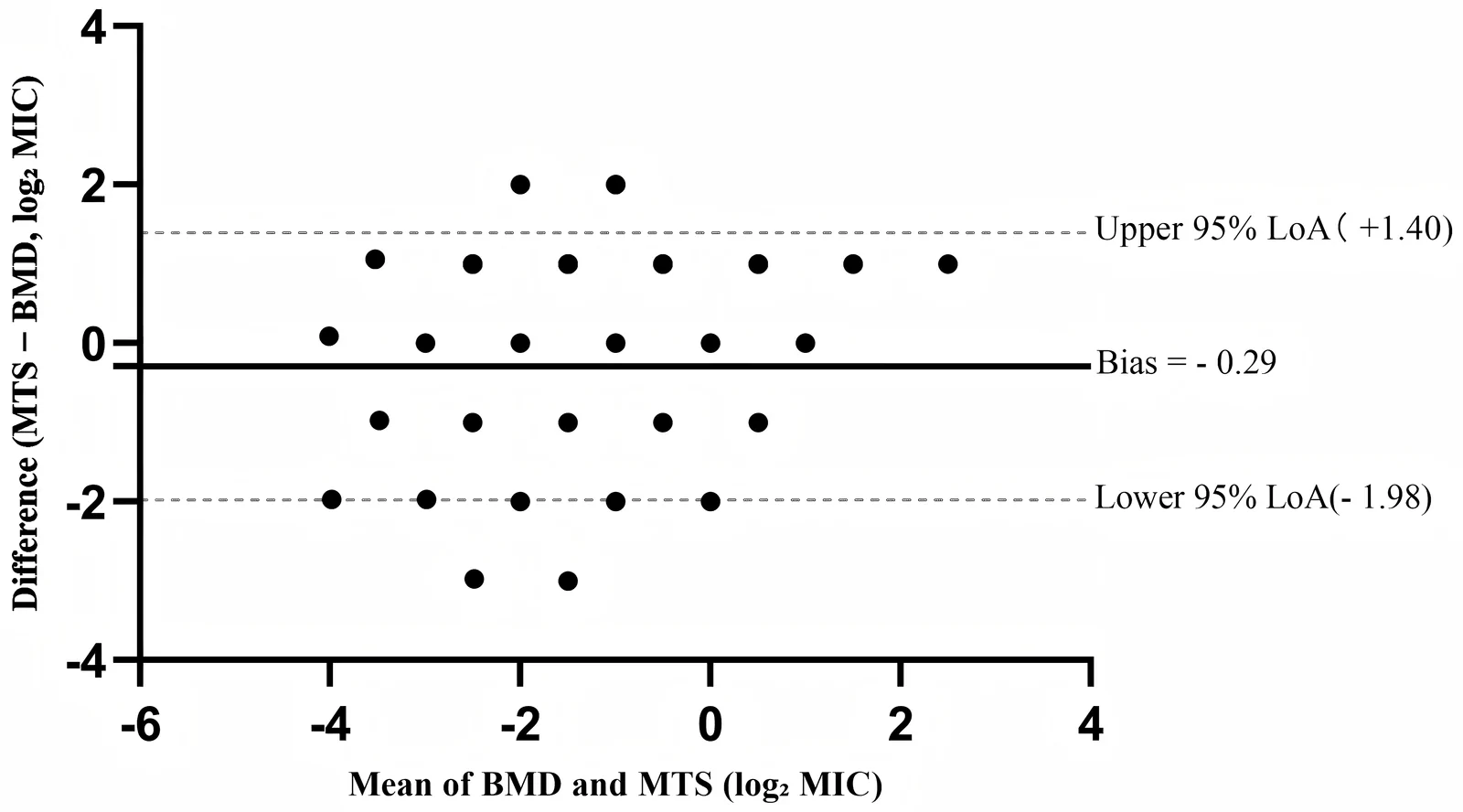
Bland-Altman plot comparing eravacycline MIC values determined by MTS and BMD (n = 345). Differences of log_2_-transformed values (MTS − BMD) are plotted against the mean of the two methods. Solid line, mean difference (bias); dashed lines, 95% limits of agreement.

**Figure 2:**
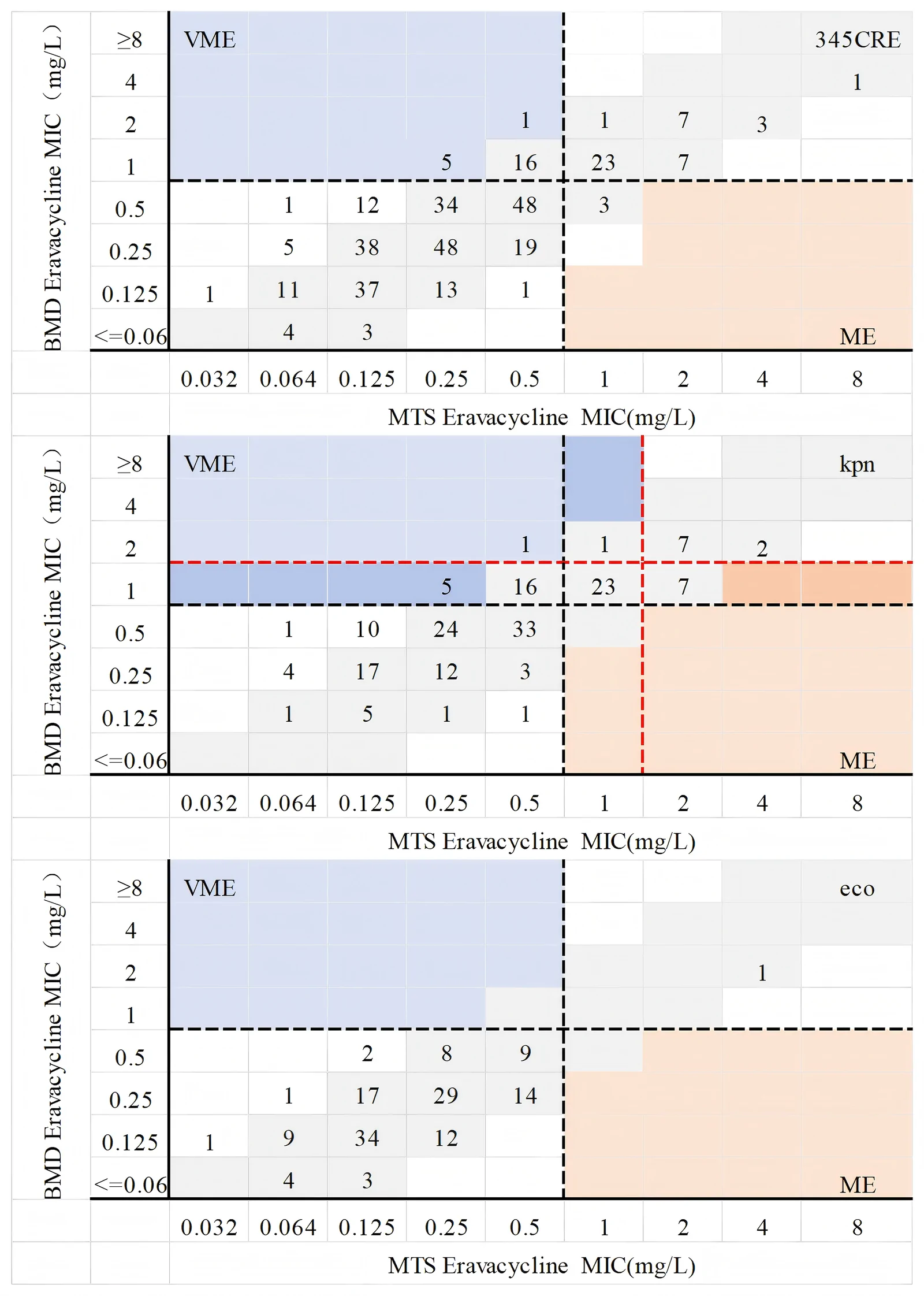
Scatter plots comparing eravacycline MICs by MTS with reference BMD for 345 CRE, 174 CRKP, and 144 CREC isolates. The black dashed line indicates the FDA susceptibility breakpoint, and the red dashed line indicates the ChinaCAST breakpoint. Gray, blue, and orange shading represent EA, VME, and ME regions, respectively.

**Table 5:** Performance of MTS for eravacycline susceptibility testing stratified by different CRE species.

Organism		FDA				ChinaCAST			
	NO.	ME	VME	EA	CA	ME	VME	EA	CA
All CRE	345	1.1% (3/280) (0.3%-3.1%)	33.8% (22/65) (23.4%-45.8%)	92.5% (319/345) (89.2%–94.8%)	92.8% (320/345) (89.5%-95.0%)	–	–	–	–
CRKP	174	0 (0-3.3%)	35.5% (22/62) (24.7%-47.9%)	87.4% (152/174) (81.6%–91.5%)	87.4% (152/174) (81.6%–91.5%)	4.3% (7/163) (2.1%-8.6%)	18.2% (2/11) (3.2%-47.7%)	87.4% (152/174) (81.6%–91.5%)	94.8% (165/174) (90.5%-97.3%)
CREC	144	0 (0-2.6%)	0 (0-79.3%)	97.2% (140/144) (93.1%-98.9%)	100.0% (144/144) (97.4%-100%)	0 (0-2.6%)	0 (0-79.3%)	97.2% (140/144) (93.1%-98.9%)	100.0% (144/144) (97.4%-100%)

ChinaCAST, Expert Committee of the National Health Commission on Antimicrobial Susceptibility Testing and Standard Research; FDA, Food and Drug Administration; “-” indicates no breakpoint has been defined for the corresponding organism and test method by the respective standardizing body; ME, major error; VME, very major error; CRKP, carbapenem-resistant *K. pneumoniae*; CREC, carbapenem-resistant *E. coli;* MTS, MIC test strip. Species-specific CA/EA/VME/ME was calculated for CRKP and CREC only (n = 318); the remaining 27 breakpoint-applicable isolates of other Enterobacterales were excluded due to limited sample sizes. 95% CIs are shown beneath each proportion.

### 3.3 Disk diffusion

DD testing yielded inhibition zone diameters ranging from 8 to 22 mm, with an overall susceptibility rate of 83.2% (287/345). Compared with BMD under FDA breakpoints, CA for all 345 CRE isolates was 89.9% (310/345), with VME of 32.3% (21/65) and ME of 5.0% (14/280). By species, CRKP exhibited CA of 82.2% (143/174), VME of 33.9% (21/62), and ME of 8.9% (10/112), whereas CREC exhibited CA of 97.9% (141/144), ME of 2.1% (3/143), and no VME. Under ChinaCAST breakpoints, CRKP showed CA of 77.0% (134/174), ME of 24.5% (40/163), and no VME; CREC showed CA of 77.8% (112/144), ME of 22.4% (32/143) and no VME (**Figure 3**, **Table 6**).

**Figure 3:**
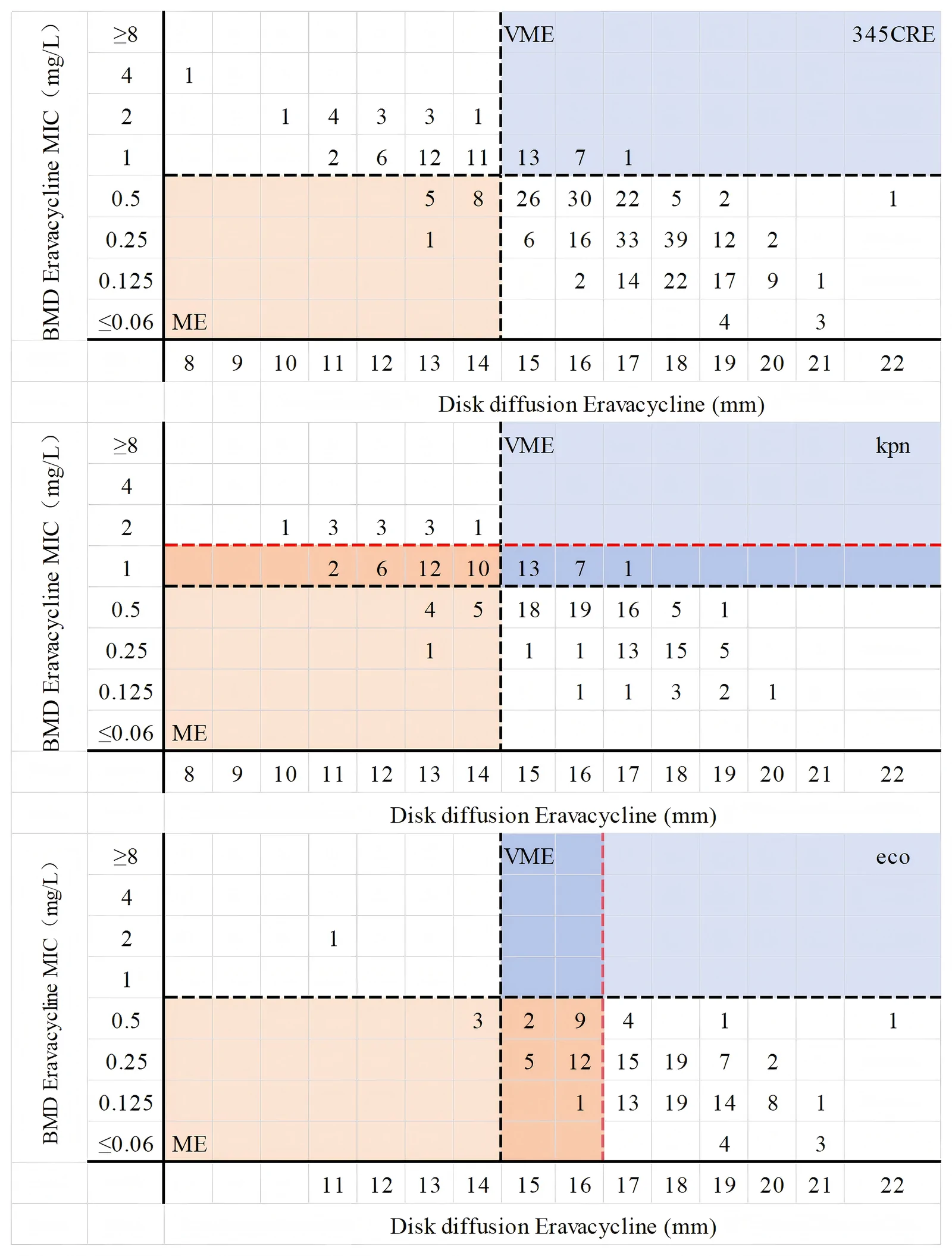
Scatter plots comparing eravacycline disk diffusion results with reference BMD for 345 CRE, 174 CRKP, and 144 CREC isolates. Black and red dashed lines denote the FDA and ChinaCAST susceptibility breakpoints, respectively. Blue and orange shading represent VME and ME regions, respectively.

**Table 6:** Performance of DD for eravacycline susceptibility testing stratified by different CRE species.

Organism		FDA			ChinaCAST		
	NO.	ME	VME	CA	ME	VME	CA
All CRE	345	5.0% (14/280) (3.0%-8.2%)	32.3% (21/65) (22.0%-44.3%)	89.9% (310/345) (86.2%-92.6%)	–	–	–
CRKP	174	8.9% (10/112) (4.9%–15.7%)	33.9% (21/62) (23.3%–46.3%)	82.2% (143/174) (75.8%–87.2%)	24.5% (40/163) (18.6%–31.7%)	0 (0–25.9%)	77.0% (134/174) (69.9%–82.9%)
CREC	144	2.1% (3/143) (0.7%–6.0%)	0 (0.0%–79.3%)	97.9% (141/144) (94.1%–99.3%)	22.4% (32/143) (16.3%–29.9%)	0 (0–79.3%)	77.8% (112/144) (70.2%–84.0%)

ChinaCAST, Expert Committee of the National Health Commission on Antimicrobial Susceptibility Testing and Standard Research; FDA, Food and Drug Administration; “-” indicates no breakpoint has been defined for the corresponding organism and test method by the respective standardizing body; ME, major error; VME, very major error;CRKP, carbapenem-resistant *K. pneumoniae*; CREC, carbapenem-resistant *E. coli;* DD, disk diffusion, Species-specific CA/EA/VME/ME was calculated for CRKP and CREC only (n = 318); the remaining 27 breakpoint-applicable isolates of other Enterobacterales were excluded due to limited sample sizes. 95% CIs are shown beneath each proportion.

## 4 Discussion

Eravacycline demonstrated potent *in vitro* activity against the 358 CRE isolates in this study. The isolate collection encompassed diverse carbapenemase types, including KPC-2, NDM-1/4/5/13/23, and KPC-2 plus NDM-1 co-producers. Eravacycline MIC distributions for these different enzyme types clustered within the 0.06-2 mg/L range, with no discernible shift in activity attributable to carbapenemase variation. This suggests that, as a ribosomal inhibitor, eravacycline retains antibacterial efficacy irrespective of common carbapenemase types (Zhang et al., 2016; Chen et al., 2023). This MIC distribution is largely consistent with recent multicenter data from China reporting MIC_50/90_ of 0.25/1 mg/L for CRE (Chen et al., 2023), as well as the activity range observed for Enterobacterales in a global 5-year surveillance network (MIC_50/90,_ 0.25/0.5 mg/L) (Morrissey et al., 2020; Hawser et al., 2023). For *E. coli*, the MIC_50/90_ (0.25/0.5 mg/L) were highly concordant with the 2,866 isolates reported by Abdallah et al. (MIC_50/90_, 0.12/0.5 mg/L) (Abdallah et al., 2015) and CHINET surveillance data from China (MIC_50/90_, 0.125/0.5 mg/L) (Yang et al., 2025); whereas for *K. pneumoniae*, the MIC_50/90_ (0.5/1 mg/L) were higher and susceptibility rate (64.4%, FDA criteria) was lower than those observed in *E. coli*, consistent with the epidemiological characteristics of CRKP in Zhejiang described by Zou et al. (susceptibility rate 53.1%, FDA criteria) (Zou et al., 2023; Yang et al., 2025). These findings further confirm that eravacycline maintains potent *in vitro* activity against predominant CRE strains circulating in China, in agreement with relevant studies both domestically and internationally. Among the 65 BMD-nonsusceptible isolates, only one carried the eravacycline-specific resistance determinant *tet*(X4), while classic tetracycline resistance genes were detected in others but are not expected to compromise eravacycline activity due to its fluorocycline structure (Sutcliffe et al., 2013; Falagas et al., 2025).

Although BMD remains the reference method for susceptibility testing, its labor-intensive procedures, stringent technical requirements, and high cost limit large-scale implementation in primary-level and routine clinical laboratories. Consequently, validation of convenient and reliable commercial alternative methods is urgently needed. Currently, published methodological validation data for eravacycline remain limited, with previous studies focusing predominantly on non-enteric organisms such as *Staphylococcus aureus* and *Acinetobacter baumannii* (Liu et al., 2025; Wang et al., 2026). Recently, Ronsin et al. provided important validation data for 303 carbapenem-non-susceptible Enterobacterales under EUCAST breakpoints (Ronsin et al., 2026); however, species-specific evaluation for *K. pneumoniae* was not performed due to the absence of EUCAST criteria, and comparative data under FDA and ChinaCAST breakpoints remain lacking. Systematic validation specifically for CRE under these clinically relevant breakpoint systems is therefore still needed. The gradient diffusion method is operationally simple, requires no specialized equipment, and is well suited for rapid susceptibility testing of individual clinical isolates. While Yang et al. (Yang et al., 2025) recently evaluated eravacycline susceptibility testing by disk diffusion, MTS, and BMD in a large multicenter Chinese collection, their study encompassed diverse species with varying resistance profiles. The present study focused specifically on CRE isolates, for which accurate susceptibility testing is most clinically critical, and directly compares FDA and ChinaCAST breakpoints within this high-risk population, providing targeted evidence for laboratories managing carbapenem-resistant infections.

Using BMD as the reference and FDA breakpoints, MTS achieved an EA of 92.5% and a CA of 92.8% among 345 CRE isolates, both meeting the ≥90% acceptability criteria specified in CLSI M52. However, the overall VME rate reached 33.8%, markedly exceeding the acceptable upper limit of ≤1.5%. After adjustment for the lack of an intermediate breakpoint, the VME rate decreased to 9.2%, indicating that the majority of crude VMEs were driven by breakpoint steepness rather than systematic method failure. Quantitatively, Bland-Altman analysis revealed a mild systematic negative bias for MTS. However, the categorical consequence of this bias is significant near breakpoints, as evidenced by the high VME rate. This instability is further amplified by the absence of an intermediate category; a susceptible-only breakpoint leaves no buffer zone to absorb one-dilution discrepancies, thereby increasing the risk of categorical misclassification. This breakpoint effect is particularly pronounced for CRKP. Jing et al. reported that the epidemiological cut-off value (ECOFF) for *K. pneumoniae* is 2 mg/L (Jing et al., 2024), substantially exceeding the FDA susceptible breakpoint (≤0.5 mg/L); the observed MIC_50/90_ of 0.5/1 mg/L in the present study places a high proportion of isolates at the critical interpretive boundary. In contrast, the ECOFF for *E. coli* (0.5 mg/L) coincides with the FDA breakpoint, and CREC MICs were well below this threshold, explaining the absence of VMEs for this species. These findings for CREC are consistent with a recent large-scale validation study by Ronsin et al (Ronsin et al., 2026), who reported acceptable performance of Liofilchem MTS (EA 98.3%) and disk diffusion (CA 100%) for *E. coli* using EUCAST breakpoints. Notably, that study did not evaluate species-specific categorical agreement for *K. pneumoniae* due to the absence of EUCAST breakpoints, a gap that the present data directly address. The VME risk and CA data (90.8%, ChinaCAST criteria) reported by Yang et al. for MTS testing of Chinese CRKP isolates (Yang et al., 2025) are consistent with our findings, further confirming that the use of ChinaCAST breakpoints yielded higher CA of MTS for detecting CRKP eravacycline susceptibility.

Error analysis showed that the overall ME rate for MTS was 1.1% in all CRE, well below the 3% acceptability limit, indicating favorable safety performance; however, VMEs were concentrated predominantly in *K. pneumoniae*. Upon applying ChinaCAST breakpoints, CA for CRKP increased to 94.8% and VME decreased to 18.2%, although ME increased to 4.3%. These observations underscore that breakpoint selection exerts a decisive influence on categorical outcomes: the FDA threshold for CRKP (≤0.5 mg/L) classifies isolates with MICs of 0.5-1 mg/L as nonsusceptible, whereas the ChinaCAST threshold (≤1 mg/L) classifies the same isolates as susceptible, thereby reducing apparent VMEs but increasing MEs (Yang et al., 2025). In summary, the gradient diffusion method satisfied CLSI M52 validation criteria for the 144 CREC isolates under both FDA and ChinaCAST breakpoints and can be applied directly in clinical practice. For the 174 CRKP isolates, although ChinaCAST breakpoints increased CA to 94.8%, ME (4.3%) and the limited number of nonsusceptible isolates precluded robust VME assessment; therefore, isolates with MTS readings in the 0.5-2 mg/L range (± 1 twofold dilution from the ChinaCAST breakpoint) should be verified by BMD prior to reporting.

Under FDA breakpoints, DD yielded an overall CA of 89.9% and a VME of 32.3% concentrated in CRKP; in contrast, CREC exhibited no VME. CRKP VMEs occurred for isolates with a BMD MIC of 1 mg/L and zones of 15-16 mm, whereas MEs occurred for isolates with a BMD MIC of 0.5 mg/L and zones of 13-14 mm. As established above, the disconnect between the *K. pneumoniae* ECOFF and FDA breakpoint compresses isolates at the threshold (Jing et al., 2024). For DD, this MIC compression translates into zone diameters of 13–16 mm, where ±1 mm variability produces categorical misclassification. Notably, the present CRKP MIC_90_ (1 mg/L) was lower than Yang et al. had reported for general clinical isolates (2 mg/L) indicating a more tightly clustered distribution that amplifies categorical errors (33.9% *versus* 6.8%) (Yang et al., 2025). In contrast, CREC MICs were well below the ECOFF/breakpoint at 0.5 mg/L (Jing et al., 2024), precluding VMEs. Under ChinaCAST breakpoints, the raised MIC threshold without recalibration of the zone breakpoint increased MEs. These DD findings for CREC align with Ronsin et al (Ronsin et al., 2026), who reported CA 100% for *E. coli* DD; however, for CRKP, neither their study nor ours supports routine DD use. Thus, DD is reliable for CREC under FDA breakpoints but should serve only as a screening tool for CRKP. Under ChinaCAST breakpoints, BMD confirmation is mandatory for both *E. coli* and *K. pneumoniae* due to elevated MEs.

This study has several limitations. First, all isolates originated from a single center, potentially introducing regional bias, so multicenter and local validation are therefore warranted. Second, the limited number of uncommon species and isolates lacking applicable breakpoints precluded robust species-specific evaluation. Third, although whole-genome sequencing was performed for carbapenemase genotyping, these data were not further analyzed for eravacycline-specific resistance mechanisms.

## 5 Conclusion

In conclusion, eravacycline demonstrated potent *in vitro* activity against clinical CRE isolates. The gradient diffusion method showed excellent concordance with reference BMD for CREC under both FDA and ChinaCAST breakpoints, making it suitable for routine testing of this species. This finding is consistent with the current EUCAST breakpoint policy, which provides eravacycline susceptibility criteria exclusively for *E. coli* among Enterobacterales, further underscoring the necessity of species-specific method validation. For CRKP, MTS cannot be recommended as a standalone routine method, and reference BMD is required for definitive susceptibility reporting. DD performed reliably for CREC under FDA breakpoints. For CRKP, accuracy was substantially lower due to the high proportion of isolates falling in the critical 13–16 mm zone near the breakpoint. Under ChinaCAST breakpoints, however, DD yielded elevated error rates for both species. BMD confirmation is mandatory for these isolates to avoid misclassification.

## Data Availability

The raw data supporting the conclusions of this article will be made available by the authors, without undue reservation.
