# Supplementary material for: Evaluation of eravacycline susceptibility testing by MIC test strip and disk diffusion against broth microdilution in carbapenem-resistant Enterobacterales
Source: Front Cell Infect Microbiol. 2026 Sep 18;16:1932734. doi: 10.3389/fcimb.2026.1932734 (PMC13630635; doi:10.3389/fcimb.2026.1932734)
Supplement: Supplementary file 1 [file Table1.doc]

Supplementary Table S1. Carbapenemase genotypes and eravacycline resistance determinants of 65 isolates categorized as eravacycline-resistant by reference broth microdilution (BMD MIC >0.5 mg/L, FDA breakpoints).

| **NO.** | **BMD(mg/L)** | **Carbapenemase** | **Species** | **Classictetracyclineresistancegene** | **Eravacycline-specificresistancegene** |
| --- | --- | --- | --- | --- | --- |
| 1 | 1 | *bla*_KPC-2_ | KPN | *tet*(A) | ND |
| 3 | 1 | *bla*_KPC-2_ | KPN | ND | ND |
| 6 | 1 | *bla*_KPC-2_ | KPN | ND | ND |
| 7 | 2 | Carbapenemase-negative | KPN | ND | ND |
| 8 | 1 | *bla*_KPC-2_ | KPN | ND | ND |
| 9 | 4 | *bla*_NDM-5_ | ECL | *tet*(A)、*tet*(D) | ND |
| 10 | 1 | *bla*_KPC-2_ | KPN | ND | ND |
| 13 | 1 | *bla*_NDM-5_ | KPN | ND | ND |
| 15 | 2 | Carbapenemase-negative | ECO | ND | *tet*(X4) |
| 16 | 1 | *bla*_KPC-2_ | KPN | ND | ND |
| 17 | 1 | *bla*_KPC-2_ | KPN | *tet*(A) | ND |
| 18 | 1 | *bla*_KPC-2_ | KPN | *tet*(A) | ND |
| 19 | 1 | *bla*_KPC-2_ | KPN | ND | ND |
| 22 | 1 | *bla*_KPC-2_ | KPN | *tet*(A) | ND |
| 23 | 1 | *bla*_KPC-2_ | KPN | *tet*(A) | ND |
| 24 | 1 | *bla*_KPC-2_ | KPN | *tet*(A) | ND |
| 29 | 1 | *bla*_KPC-2_ | KPN | ND | ND |
| 32 | 1 | *bla*_KPC-2_ | KPN | ND | ND |
| 35 | 1 | *bla*_KPC-2_ | KPN | *tet*(A) | ND |
| 37 | 2 | Carbapenemase-negative | KPN | *tet*(A) | ND |
| 42 | 2 | *bla*_KPC-2_ | KPN | ND | ND |
| 43 | 2 | *bla*_KPC-2_ | KPN | ND | ND |
| 44 | 1 | *bla*_KPC-2_ | KPN | ND | ND |
| 47 | 1 | Carbapenemase-negative | KPN | ND | ND |
| 48 | 1 | *bla*_KPC-2_＋bla_NDM-1_ | KPN | ND | ND |
| 49 | 2 | *bla*_KPC-2_ | KPN | ND | ND |
| 57 | 1 | *bla*_KPC-2_ | KPN | ND | ND |
| 59 | 1 | *bla*_KPC-2_＋bla_NDM-1_ | KPN | ND | ND |
| 61 | 1 | *bla*_KPC-2_ | KPN | *tet*(A) | ND |
| 88 | 2 | *bla*_KPC-2_ | KPN | *tet*(A) | ND |
| 89 | 2 | *bla*_KPC-2_ | KPN | *tet*(A) | ND |
| 94 | 1 | Carbapenemase-negative | KPN | *tet*(D) | ND |
| 99 | 1 | *bla*_KPC-2_ | KPN | ND | ND |
| 101 | 1 | *bla*_KPC-2_ | KPN | *tet*(A) | ND |
| 110 | 2 | *bla*_KPC-2_ | KPN | ND | ND |
| 111 | 1 | *bla*_KPC-2_ | KPN | ND | ND |
| 113 | 1 | Carbapenemase-negative | KPN | ND | ND |
| 117 | 1 | *bla*_NDM-1_ | KPN | ND | ND |
| 123 | 1 | *bla*_NDM-1_ | ECL | ND | ND |
| 124 | 2 | *bla*_KPC-3_ | KPN | *tet*(A) | ND |
| 125 | 1 | *bla*_KPC-2_ | KPN | ND | ND |
| 140 | 1 | *bla*_KPC-2_ | KPN | ND | ND |
| 155 | 1 | *bla*_KPC-2_ | KPN | ND | ND |
| 159 | 1 | *bla*_KPC-2_ | KPN | *tet*(A) | ND |
| 160 | 1 | *bla*_KPC-2_ | KPN | ND | ND |
| 162 | 1 | *bla*_KPC-2_ | KPN | ND | ND |
| 165 | 2 | *bla*_NDM-5_ | KPN | ND | ND |
| 183 | 1 | *bla*_KPC-2_ | KPN | ND | ND |
| 186 | 1 | *bla*_KPC-2_ | KPN | ND | ND |
| 198 | 1 | *bla*_KPC-2_ | KPN | ND | ND |
| 199 | 1 | *bla*_KPC-2_ | KPN | *tet*(A) | ND |
| 208 | 1 | Carbapenemase-negative | KPN | *tet*(A) | ND |
| 212 | 1 | *bla*_KPC-2_ | KPN | *tet*(A) | ND |
| 214 | 1 | *bla*_KPC-2_ | KPN | *tet*(A) | ND |
| 218 | 1 | *bla*_KPC-2_ | KPN | *tet*(A) | ND |
| 221 | 1 | Carbapenemase-negative | KPN | ND | ND |
| 222 | 1 | Carbapenemase-negative | KPN | *tet*(A) | ND |
| 223 | 2 | *bla*_NDM-23_ | KPN | *tet*(A) | ND |
| 225 | 1 | *bla*_KPC-2_ | KPN | ND | ND |
| 226 | 1 | *bla*_KPC-2_ | KPN | *tet*(A) | ND |
| 227 | 1 | Carbapenemase-negative | KPN | *tet*(A) | ND |
| 228 | 1 | *bla*_KPC-2_ | KPN | *tet*(A) | ND |
| 237 | 1 | *bla*_KPC-2_＋bla_NDM-1_ | KPN | *tet*(A) | ND |
| 255 | 1 | *bla*_KPC-2_ | KPN | *tet*(A) | ND |
| 263 | 1 | *bla*_KPC-2_ | KPN | *tet*(A) | ND |

ND, not detected. *tet*(X4) is the only eravacycline-specific resistance gene detected (1/65 isolates). Classic tet genes (*tet*(A), *tet*(D)) do not confer eravacycline resistance due to its fluorocycline structure.
